# The Targeting of Nuclear Factor Kappa B by Drugs Adopted for the Prevention and Treatment of Preeclampsia

**DOI:** 10.3390/ijms23052881

**Published:** 2022-03-07

**Authors:** Agata Sakowicz

**Affiliations:** Department of Medical Biotechnology, Medical University of Lodz, 90-752 Lodz, Poland; agata.sakowicz@gmail.com; Tel.: +48-502-615-332

**Keywords:** Nuclear Factor kappa B, preeclampsia, prevention of preeclampsia, treatment

## Abstract

Preeclampsia (PE) is characterised by high levels and activity of the transcription factor Nuclear Factor kappa B (NFĸB) in the maternal blood and placental cells. This factor is responsible for the regulation of over 400 genes known to influence processes related to inflammation, apoptosis and angiogenesis, and cellular responses to oxidative stress and hypoxia. Although high NFĸB activity induces hypoxia and inflammation, which are beneficial for the process of implantation, NFĸB level should be reduced in the later stages of physiological pregnancy to favour maternal immunosuppression and maintain gestation. It is believed that the downregulation of NFĸB activity by pharmacotherapy might be a promising way to treat preeclampsia. Interestingly, many of the drugs adopted for the prevention and treatment of preeclampsia have been found to regulate NFĸB activity. Despite this, further innovation is urgently needed to ensure treatment safety and efficacy. The present article summarizes the current state of knowledge about the drugs recommended by cardiology, obstetrics, and gynaecology societies for the prevention and treatment of preeclampsia with regard to their impact on the cellular regulation of NFĸB pathways.

## 1. Introduction

Preeclampsia (PE) is responsible for 5–10% of pregnancy complications and is recognized as one of the most common reasons for maternal and foetal death. While it generally appears after week 20 of pregnancy, cases with very early (i.e., before week 20) and very late symptoms (i.e., in the first 6 weeks after birth) have been reported [1,2]. The guidelines presented by international obstetrics and gynaecology societies characterise preeclampsia as the sudden occurrence of hypertension (i.e., >140 mmHg systolic or >90 mmHg diastolic) in previously normotensive women, accompanied by proteinuria (i.e., >300 mg for 24 h or at least 2+ on a dipstick). Preeclampsia can also be recognised by the presence of hypertension complicated by at least one of the following symptoms: serum creatinine level >1 mg/dL, elevated transaminase levels, thrombocytopenia, haemolysis, neurological disorders, or uteroplacental dysfunction (i.e., foetal growth restriction) [3,4].

A considerable body of evidence indicates that blood from patients with preeclampsia demonstrates a high level of inflammatory factors (such as: TNF-α, IFN-γ, IL6 or IL1) and reactive oxygen species (ROS), and that preeclamptic placentas display features indicative of chronic hypoxia throughout the gestation period [5,6,7]. Inflammation, oxidative stress, ineffective angiogenesis, and hypoxia are regulated at the cellular level by numerous pathways, most of which are under the control of nuclear factor kappa B (NFĸB) [8]. NFĸB level and activity are both upregulated in maternal and placental cells, and as this upregulation is strongly linked with the pathomechanism of preeclampsia, it is possible that preeclampsia could be treated by drugs targeting the mechanism of NFĸB activation. Interestingly a number of agents already adopted for the prophylaxis and treatment of preeclampsia are believed to modulate NFĸB activity. This article reviews the current state of knowledge about the cellular mechanism of action of drugs recommended by international societies for prevention and treatment of preeclampsia with regard to their influence on NFĸB.

## 2. NFĸB and Its Relationship with Preeclampsia Development

To prepare for pregnancy, the endometrium demonstrates an extensive increase in NFĸB expression to prepare maternal tissues for the opening of the implantation window in the case of fertilization [9,10,11], and this NFĸB activation continues in the uterus during the implantation period [8,9,10]. NFĸB activation affects the regulation of inflammatory factors, such as TNFα, IL6, IL8, and IFN-γ, secreted by the endometrial cells, as well as by the natural killer cells (NK), macrophages, dendritic cells, or lymphocytes of the maternal immune system that are recruited to the site of implantation [7].

Multiple pieces of evidence emphasize the role of inflammation in the regulation of trophoblast migration and invasion [7,12]. Interestingly, the same pro-inflammatory factors (e.g., TNFα) are perceived by some studies as stimulators of trophoblast migration and invasion, and as inhibitors by others [13,14,15]. It is possible that the intensity of inflammatory reaction, related to the strength of NFĸB activation, might play a significant role in the success of implantation. The rise in the levels of proinflammatory cytokines may disturb the delicate inflammatory reactions at the feto–maternal interface during implantation, leading to its failure or pregnancy loss [16]. Moreover, such abnormal maternal inflammation has been also found to impair the remodeling of uterine spiral arteries and alter uteroplacental perfusion, leading to the development of features of preeclampsia in a rat model [17]. Abnormal, shallow placentation forces trophoblastic cells to live under hypoxic conditions, which generates oxidative stress and intensifies the inflammatory reaction. These processes are strongly influenced by the presence of NFĸB, whose level and activity is downregulated over the course of a non-complicated gestation. In preeclampsia, NFĸB level and activity are significantly elevated in the maternal blood and have been found to be up to 10-fold higher in placentas than controls [18,19].

Interestingly, although preeclampsia is characterised by elevated NFĸB activity, its mechanism of activation remains poorly explored. It is postulated that oxidative stress favours the degradation of NFĸB inhibitors in lymphocytes and aortic endothelial cells by proteosomes [20,21]; however, no such findings have been noted in human umbilical vein endothelial cells or preeclamptic placental samples [22,23].

Unstimulated cells demonstrate only basal levels of NFĸB in the cytoplasm. This level is maintained by various inhibitors, of which IĸBα (NF-Kappa B Inhibitor Alpha) and IĸBβ (NF-Kappa B Inhibitor Beta) are the most common. These bind to NFĸB and prevent its activation i.e., its phosphorylation and translocation into nucleus. However, in environments rich in reactive oxygen species or cytokines, NFĸB activation takes place, driven by various NFĸB activators. Among these, IKKα (Inhibitor of Nuclear Factor Kappa B Kinase Subunit Alpha), IKKβ (Inhibitor of Nuclear Factor Kappa B Kinase Subunit Beta), IKKγ (Inhibitor of Nuclear Factor Kappa B Kinase Subunit Gamma), and CK2 (Casein kinase 2) are implicated in the three most widely-studied pathways: canonical, non-canonical, and atypical [24].

Interestingly, in preeclamptic placentas, the canonical, non-canonical, and atypical activation pathways do not seem to play a significant role in the process of NFĸB activation. In the canonical pathway, various NFĸB activators (i.e., IKKα, IKKβ and IKKγ) are downregulated, whereas the inhibitors (i.e., IĸBα, IĸBβ) are upregulated [22,25]. Similarly, NFĸB activators participating in the non-canonical (i.e., IKKα) and atypical pathways (i.e., CK2) are also downregulated. This suggests that preeclamptic placentas employ specific NFĸB activation mechanics: their activity is independent of IKKα, IKKβ, IKKγ, and CK2, and avoids the cytoplasmic and proteosomal degradation by NFĸB inhibitors such as IĸBα or IĸBβ. Some studies suggest that this mechanism may be dependent on the activity of a p53/RSK1 (Tumour Protein p53/Ribosomal Protein S6 Kinase A1) complex [22,24].

Independently of the molecular mechanism of NFĸB activation, this factor is strongly linked with oxidative stress and inflammation. Under such conditions, placental cells secrete a range of proteins controlling vascular function, such as arginase II, endothelin-1 or soluble fms-like tyrosine kinase 1 (sFlt-1), and undergo apoptosis, shedding apoptotic debris into the maternal circulation. All of these factors contribute to maternal endothelial dysfunction, which the main cause of clinical symptoms in patients with preeclampsia [26,27,28,29,30,31].

## 3. Targeting NFĸB by Aspirin

Numerous randomised controlled clinical trials and big data meta-analyses indicate that early supplementation with low doses of aspirin, i.e., acetylsalicylic acid (ASA), is effective in preventing preeclampsia. However, although aspirin treatment improves the outcome of pregnancy, reducing the risk of preterm preeclampsia by approximately 30–62%, conflicting data exists regarding the optimal dose and time of initiation of ASA intake [32,33,34]. In numerous countries, the guidelines prepared by cardiology, gynaecology and obstetrics societies recommend the initiation of aspirin treatment before week 20 (optimum before week 16) of gestation among high- and moderate-risk women (Table 1).

After oral consumption, aspirin is quickly absorbed in the stomach and hydrolysed into salicylic acid in the intestinal circulation. The distribution of the drug peaks 30 min from oral administration. The half-life of aspirin varies between species with the 13–31 min in human subjects. Aspirin is most widely known for inhibiting arachidonic acid, which is converted into prostaglandins and thromboxane A2 (TXA2) by cyclooxygenase 1 (COX 1) or cyclooxygenase 2 (COX 2). The former is a constitutive enzyme, while the latter an inducible by inflammatory reaction, hypoxia, or oxidative stress enzyme [47].

In preeclampsia, the presence of free radicals, cytokines, and sFlt-1 influences endothelial dysfunction and COX activation, shifting the PGI2/TXA2 ratio in favour of TXA2 production [47]. It is believed that aspirin treatment lowers thromboxane production by inhibiting COX activity. However, its action is much broader: it may well modify the cellular response, influencing NFĸB regulation pathways. Experiments conducted on lipopolysaccharide (LPS)-stimulated human and mouse lymphocyte cell lines, such as Jurkat T and PD-31, indicate that salicylate supplementation of the cell medium inhibited the expression of genes regulated by NFĸB in these cells. Interestingly, while the concentration of NFĸB in the cells was unchanged, it demonstrated lower binding efficiency to the DNA responsive element, with this effect being dependent on salicylate dose [48]. This might account for the dual nature of ASA in the regulation of COX metabolism in preeclampsia. It works as a direct inhibitor of COX activity, thus maintaining a PIG2/TXA2 balance typical of non-complicated pregnancy, and as a regulator of *COX2* gene expression by kappa B factor; NFĸB response elements are present in the promotors of *PTGS2* genes coding for COX 2 [49,50].

A significant body of evidence indicates that aspirin may be an efficient agent in the inhibition of inflammatory reaction. ASA administration results in the downregulation of the transcription of the pro-inflammatory cytokines TNFα, IFN-γ, IL1, IL6, and IL8, whose genes are regulated by NFĸB [48]; all of these cytokines are strongly linked with the pathomechanism of preeclampsia. It is possible that oral administration of ASA before 16 weeks of gestation effectively inhibits the secretion of these factors by maternal immunological cells, enabling a switch from a pro-inflammatory cytokine profile, associated with Th1 and Th17 cells, to an anti-inflammatory one, associated with Th2 lymphocytes. This switch is desirable for maintaining the correct course of pregnancy [51]. This anti-inflammatory activity of salicylates is linked with the regulation of NFĸB activity; indeed, aspirin prevents NFĸB nuclear translocation and its consequent binding to the motif element on DNA [52,53]. Alternatively, it was also found that aspirin serves as a competitive inhibitor of ATP, binding to the IKKβ protein, one of the activators of NFĸB in the canonical pathway [54]. Following its inactivation, IKKβ cannot activate NFĸB, thus inhibiting its nuclear translocation [49,54].

Extensive inflammatory reactions are considered to play a central role in the development of the clinical symptoms of preeclampsia. Damaged endothelial cells expose adhesion molecules such as vascular cellular adhesion molecule-1 (VCAM-1), intracellular adhesion molecule-1 (ICAM-1) and E-selectin on their surface, and the soluble forms of these molecules have been found to be elevated in the maternal blood of preeclamptic women [55,56]. In human umbilical vein endothelial cells (HUVECs), aspirin treatment has been found to counter the mechanism of NFĸB-binding with DNA in response to TNFα. This dose-dependent inhibition was related to a reduction in the expression of VCAM-1 and E-selectin; indeed, the NFĸB binding motif is present in the genes coding for these molecules [57]. Similarly, salicylate has been found to have an inhibitory effect on the expression of VCAM-1 and ICAM-1 [58]; salicylate appears to attenuate the translocation of NFĸB into the nucleus by inhibiting the phosphorylation and subsequent degradation of kappa B inhibitor i.e., IĸBα, leading to a depletion in VCAM-1 and ICAM-1 on the cell surface [58].

Although aspirin is known to exert an influence on endothelial cells via NFĸB pathways, little is known of its ability to target NFĸB in other types of cells, especially placental cells. The NICHD Human Placental Project results suggest that oral administration of aspirin according to ACOG recommendations might correct the thromboxane/prostacyclin imbalance in the placenta. It has also been proposed that low-dose aspirin therapy might inhibit lipid peroxides in both the maternal circulation and placenta [59]. While the association between ASA and thromboxane production inhibition, and placental oxidative stress, has been examined elsewhere [60], little is known about the molecular mechanism of NFĸB regulation by ASA in human placenta. Some information in this area might be delivered by in vitro and animal studies. In the study on a preeclampsia-like mouse model after lipopolysaccharide (LPS) treatment, aspirin was found to suppress placentae NFĸB activation by downregulating the phosphorylation of the proteins implicated in the kappa B regulation pathways i.e., IKKβ and IĸBα [61]. Similarly, studies of trophoblastic HTR8/SVneo cells stimulated by sFlt-1 followed by aspirin treatment found ASA to inhibit NFĸB phosphorylation by inactivating its activators i.e., IKKα and IKKβ [62]. Panagogdage et al. [63] examined the regulation of 84 genes related to apoptosis following stimulation with 25% maternal serum and treatment with ASA in a human trophoblastic cell line (BeWo). The cells incubated with serum from preeclamptic mothers (PE) with a low dose of ASA (PE+LDA) demonstrated a significant reduction in *IKBKE* (Inhibitor of Nuclear Factor Kappa B Kinase Subunit Epsilon) and *p53* gene expression compared to cells treated with PE serum alone. However, no significant differences were observed between PE+LDA cells and cells incubated with serum from normotensive pregnant women [63]. It is possible, then, that ASA may have some impact on the regulation of NFĸB activation pathways in placental cells in preeclampsia. Both IKKε (Inhibitor of Nuclear Factor Kappa B Kinase Subunit Alpha Epsilon) coded by *IKBKE*, and the p53 protein are known to influence NFĸB activation [64,65]. The active form of IKKε promotes NFĸB activation and its transcriptional activity [64], whereas p53 influences the presence of the active form of NFĸB (i.e., phospho-NFĸB at Ser536) in the nucleus of trophoblastic cells under preeclamptic conditions [24]. However, further studies are needed to fully understand the effect of aspirin on the p53-dependent NFĸB activation pathway in placental cells. This pathway seems to be important for the development of preeclampsia [22,24] and therefore might be a good therapeutic target in the treatment of this disease.

As a number of embryonic processes taking place during placenta formation are linked to cancer development [66], it is possible that cancer and placental cells might demonstrate a similar response to aspirin treatment. HeLa cells were found to be sensitized to TNFα by aspirin, resulting in apoptosis, and this action was directly related to the repression of NFĸB [67]. Moreover, supplementation of cell culture medium by aspirin in an environment rich in TNFα and IL6 or reactive oxygen species (ROS) reduced the phosphorylation and/or degradation of NFĸB inhibitors i.e., IĸBα and IĸBβ [67,68].

Surprisingly, other studies found aspirin as an inductor of NFĸB activation in colon cancer cells; aspirin treatment was associated with the augmented degradation of kappa B inhibitor i.e., IĸBα protein and the induction of apoptosis [69,70,71]. Similar results were observed for tumour tissue obtained from mice bearing the xenografted HT-29 cells and for intestinal adenomas cells obtained from mice heterozygous for APC^Min+/−^ C57BL/6. In both cases, aspirin supplementation resulted in an increase in phosphorylation and IĸBα degradation, the latter supporting NFĸB nuclear translocation [72]. Surprisingly, aspirin-mediated NFĸB nuclear translocation repressed NFĸB-driven reported gene activity rather than supporting it [71,72].

Despite the significant body of evidence indicating the role of aspirin in the regulation of NFĸB activation and transcription, the data regarding the mechanism of ASA action on NFĸB activation pathways remains inconsistent. It is possible that such NFĸB regulation depends on cell type, salicylate dose, and various environmental factors. The intrauterine environment is known to change over the course of pregnancy, together with a number of pathways taking place in placental cells; as such, it is possible that the mechanism of action of aspirin on NFĸB activation may also vary depending on the stage of gestation. This might explain why studies on the benefits of ASA oral supplementation in pregnancy recommend different doses and times of treatment initiation to reduce the risk of preeclampsia.

## 4. Antihypertensive Therapy Modulates the NFĸB Pathway

According to the Global Burden of Diseases, Injuries and Risk Factors Study 2019, hypertensive disorder of pregnancy increased by over 10% between 1990 and 2019 [73]. Among these, more than half of women required treatment with one antihypertensive drug, while 8–40% of hypertensive cases required more than one [74]. Monotherapy with beta-blockers (e.g., labetalol or metoprolol or oxprenolol) or alpha-2 adrenergic receptor agonists (e.g., methyldopa) is widely accepted as a first line treatment of hypertension in pregnancy. The calcium channel blocker nifedipine is also permitted for treating pregnancy hypertension by many obstetrics and gynaecology societies. Hydralazine, a direct vasodilator, was also included in the treatment of acute severe hypertension; however, it should be carefully managed due to the occurrence of hypotension [74]. It is recommended that both angiotensin-convertase inhibitors (ACE) and adrenergic receptor blockers (ADR) should be avoided before and during pregnancy [39,75,76].

Although the influence of drugs adopted for treatment of preeclampsia on blood pressure is well understood, their precise activity at the cellular level remains unknown. Interestingly, all groups of drugs (i.e., agonist alpha-2 adrenergic receptors, beta-blockers as well as calcium channel blocker) approved for the treatment of pregnancy hypertension directly or indirectly modulate the NFĸB activation pathway.

### 4.1. Agonist of Alpha-2 Adrenergic Receptors and NFĸB

Methyldopa is an agonist of alpha-2 adrenergic receptors, which inhibit vasoconstriction and down-regulate catecholamine levels in the blood. As this drug regulates blood pressure without affecting the maternal uterine artery, it does not appear to have any negative impact on uteroplacental circulation or foetal growth [77]. Alpha-2 adrenergic receptor agonists were found to influence the transcriptional activity of NFĸB in PC12 cell line. Although its precise mechanism has never been studied, it has been suggested that alpha-2 adrenergic receptor activation induces the transcriptional nature of NFĸB via phosphoinositide-3-kinase (PI3K) [78], a known upstream regulator of NFĸB [7,79].

### 4.2. Beta-Blockers Modulate NFĸB Activation Pathways

Labetalol representing beta-blockers is widely used as a first-line antihypertensive drug during pregnancy. It non-selectively antagonizes both beta-1 and beta-2 adrenergic receptors and also works as a selective inhibitor of alpha-adrenergic receptors. Interestingly, the activation of beta-2 adrenergic receptors downregulates NFĸB activation in HUVEC cells, by lowering the level of the active (phosphorylated at Serine 536) form of NFĸB in the nucleus. This nuclear depletion was related to the inhibition of phosphorylation and proteosomal degradation of kappa B inhibitor i.e., IĸBα. This effect was totally reversed by beta-blockers [80], which might suggest that beta-blockers act as strong inductors of NFĸB transcriptional activity in HUVEC cells.

However, beta-blockers were found to have an opposite effect in a study on human peripheral blood T cells. Lymphocytes isolated from blood were stimulated by various factors in the presence or absence of beta-blockers in the cell medium. Exposure to beta-blockers resulted in the attenuation of NFĸB transcriptional activity. This effect was related to the dysregulation of nuclear translocation of NFĸB following inhibition of IKKα, known to activate kappa B via the canonical and non-canonical pathways. It is important to note that not all tested beta-blockers demonstrated this inhibitory effect, including labetalol [81].

### 4.3. Calcium Channel Blockers Inhibit the NFĸB Activation Pathways

Calcium antagonists, i.e., calcium channel blockers including nifedipine, are recommended by numerous cardiologic, genecology, and obstetrician societies for the treatment of hypertension in pregnancy [39,44,75,82]. The ACOG Committee recommends the immediate release of nifedipine as a first-line drug for severe intrapartum or postpartum hypertension treatment [82]; however, the Society of Obstetric Medicine of Australian and New Zealand (SOMANZ) proposes that nifedipine should be considered as a second-line agent for lowering blood pressure in preeclampsia and for treating gestational or chronic hypertension [76].

Calcium channel blockers work by inhibiting calcium influx into vascular smooth muscle cells, resulting in arterial vasodilation and reduction of systemic vascular resistance [74,77]. Numerous in vitro studies indicate that calcium channel blockers participate in the suppression of immune reaction via the inhibition of activity of lymphocytes, macrophages or mast cells [83]. Interestingly, this function is probably realized via regulation of NFĸB activation pathways. In a human epithelium-like lung carcinoma cell line, nifedipine was found to significantly inhibit the NFĸB/DNA binding reaction, which may partly explain its immunosuppressive effect. Moreover, this effect was dependent on nifedipine dose, with stronger inhibition of NFĸB activity being observed at higher drug concentrations [83].

Another study showed that after oral administration, nifedipine effectively decreases inflammation and increases the function of endothelial cells in coronary circulation [84]. This anti-inflammatory effect of nifedipine was also observed in hypoxic conditions. Nifedipine did not only significantly reduce the level of inflammatory factors in the lung homogenate of rats exposed to hypoxia but also down-regulated the nuclear presence of NFĸB in the studied cells. No change in NFĸB activator level (IKKα) was observed, but a significant role appears to be played by attenuation of phosphorylation followed by degradation of the NFĸB inhibitor [85]. In preeclampsia, nifedipine was also found as a negative regulator of inflammatory reaction in endothelial cells after their exposition on trophoblast debris. The expression of adhesion molecules i.e., ICAM-1 and Selectin-E on the surface of endothelial cells was found to increase after exposure to trophoblastic debris obtained from placental explants or maternal preeclamptic serum; however, nifedipine significantly reduced the level of adhesion molecules as well as inflammatory factors (i.e., IL6 and TGFβ1) secreted by endothelial cells after stimulation. It should be noted that the molecular mechanism was not precisely examined [86]. It is possible that the positive effect on endothelial function is realised by depletion of NFĸB activity, as the genes coding for ICAM-1, Selectine-E, IL6, and TGFβ1 include the binding motif for NFĸB in their promoter regions.

### 4.4. Hydralazine and Its Association with NFĸB

The most controversial drug adopted for treating hypertension in pregnancy is hydralazine, as its use is associated with various adverse effects during pregnancy. Therefore, it is included as a second-line agent, when the other drugs have failed to achieve adequate blood pressure [39,75,76]. This drug is admitted as a first-line medication in the management of severe intrapartum or postpartum hypertension according to ACOG Committee recommendations [82]. Although its mode of action and association with the NFĸB is still unclear, it was found to negatively regulate NFĸB expression in a human fibroblast cell line [87]. However, in contrast, hydrazine treatment does not appear to influence the concentration or activity of NFĸB in kidney and heart tissue from rats harbouring both human renin and angiotensin genes [88], nor modify NFĸB activation pathways in human endothelial cells after stimulation with toxic concentrations of oxyLDL [89].

## 5. Targeting NFĸB by Magnesium Sulphate Adopted for Prevention of Neurological Complication of Preeclampsia

Some of the most dangerous symptoms accompanying hypertension in preeclamptic women are associated with neurological disorders. Magnesium sulphate appears to demonstrate efficacy in the prevention of seizures in severe preeclamptic or eclamptic women. However, although moderately elevated serum magnesium is linked to a reduction of blood pressure [90], this drug is not recommended for hypertension treatment [3]. The seizure prophylaxis involving magnesium salt should be initiated in women with gestational hypertension with severe features or preeclampsia with severe features or eclampsia [3]. Magnesium sulphate is known to have an inhibitory effect on neural synapses; however, in addition to being a competitive antagonist to calcium, magnesium also controls over 300 enzymatic reactions, including those associated with glucose utilisation, protein, or nucleic acid synthesis [90]. In addition, this drug is frequently linked with NFĸB activity and immunosuppression. The administration of magnesium sulphate to pregnant rats exposed to LPS was associated with a reduction of NFĸB level and its activity in the brain of the foetus. This anti-NFĸB property of magnesium was linked with the attenuation of foetal brain inflammation [91]. Similar results were also observed by others. In primary microglia cells from Sprague–Dawley rats, the secretion of prostaglandin (PGE2), IL1β, and TNF-α was suppressed after incubation with LPS in the presence of magnesium sulphate; this was also associated with lowered NFĸB activity. Magnesium sulphate inhibits NFĸB nuclear translocation, and thus its binding capacity to DNA, in a dose dependent-manner after exposure to LPS [92].

Magnesium sulphate was also found to downregulate the inflammatory reaction in intrapartum women, term, and preterm neonates [93]. Monocytes isolated from umbilical cord blood and peripheral blood demonstrated lowered cytokine production (i.e., TNFα and IL6) after stimulation by LPS in the presence of magnesium sulphate. Magnesium inhibited the degradation of IĸBα and thus increased its level in the cells; hence, monocytes exposed to magnesium salt presented low nuclear concentrations of phosphorylated NFĸB after incubation with LPS [93]. Moreover, in a rat model obtained after surgical reduction of uterine perfusion pressure (RUPP), magnesium sulphate treatment has been found to prevent placental ischemia-induced cerebral oedema and to reduce levels of cytokines/chemokines in the cerebrospinal fluid [94]. A similar reduction in cytokine levels was also observed in the placenta of an intrahepatic cholestasis rat model as well as in human placental tissue perfused with LPS after magnesium sulphate supplementation [95,96]. The latter study indicates that magnesium salt treatment results in the attenuation of excessive placental inflammatory response in an NFĸB-dependent manner [96].

## 6. Directions for Future Research into the Treatment of Preeclampsia

Some evidence indicates that high NFĸB activity is needed before pregnancy, and at its beginning, for the success of implantation. Before pregnancy, the body is prepared by high NFĸB activation; this has been demonstrated in the uterus of non-pregnant animals and in human endometrium obtained from the perimenstrual phase of the menstrual cycle [9,10,11]. This form of activation seems to be based around the canonical pathway [11]. After conception, in the first trimester of pregnancy, NFĸB activation is continued in the decidua to support implantation; however, during this time, the canonical pathway appears to be superseded by the non-canonical one [11,51].

It is possible that any reduction of NFĸB activity before the opening of the implantation window by drugs such as aspirin, or during this period, might worsen the outcome of pregnancy. For example, some studies indicate a positive correlation between aspirin use around conception and miscarriage [97]; however, it has also been reported that aspirin use in the first lunar month of pregnancy did not increase the risk of adverse pregnancy outcome [98]. Numerous obstetrics, gynaecology, and cardiology societies propose that the best time for supplementation of ASA, i.e., to get the greatest benefit for the correct course of pregnancy, is between weeks 12–16 of gestation.

It is possible that at the turn of the first and second trimester, the non-canonical NFĸB activation pathway still dominates. As such, being an inhibitor of IKKα (the non-canonical activator of NFĸB), ASA would support the suppression of the inflammatory reaction. Indeed, in a non-complicated pregnancy, the inflammatory reaction calms during weeks 12–16; this is associated with a shift in the maternal immunological response from Th1 toward Th2 and the transformation of myometrial segments of spiral arteries [99,100]. Both processes are mediated by NFĸB, and any disturbances in them are strongly linked with the pathomechanism of preeclampsia [7,51,101].

After week of 16 of gestation, pharmacological inhibition of NFĸB might not be so effective; the prolonged, elevated NFĸB activity results in changes in cellular pathways, the appearance of a new NFĸB activation mechanism (e.g., dependent on the p53/RSK1 complex), which is insensitive to the drug, or both. This might explain the observations that aspirin treatment after week 16 is less beneficial and results in only a modest reduction of preeclampsia risk, or none at all [102]. It could also explain why NFĸB-targeting drugs used for the treatment of preeclampsia are not effective enough to eliminate the symptoms of preeclampsia and to restore the correct course of pregnancy.

Therefore, there is a need to more closely examine the mechanisms of NFĸB activation in maternal and placental cells under unfavorable conditions; these pathways might be important targets for interventions aimed at preventing preeclampsia, even though they change with the course of gestation. The use of aspirin between weeks of 12–16 of pregnancy should be maintained according to obstetrician, gynaecological, and cardiologic societies’ recommendations. However, in women at high risk of preeclampsia, where treatment is initiated after week 16, the dosage of aspirin should be supported by other, as yet unknown, drugs aimed at silencing the predominant mechanism of NFĸB activation characteristic of this phase of pregnancy. Similarly, although the drugs used for the treatment of preeclampsia are believed to generally influence NFĸB activity, they do not target the predominant NFĸB activation pathway active in preeclamptic pregnancy at the time of treatment initiation. As such, caesarian section is still recognized as a the gold standard in treatment of preeclampsia.

## 7. Conclusions

A significant body of evidence indicates that NFĸB plays a role in the development of preeclampsia. It is recognised as a key driver of the augmented inflammatory reaction at all three stages of preeclamptic pregnancy. High NFĸB levels and intensified activity have been observed in maternal blood and in the placentas of women suffering from preeclampsia.

**Figure 1:**
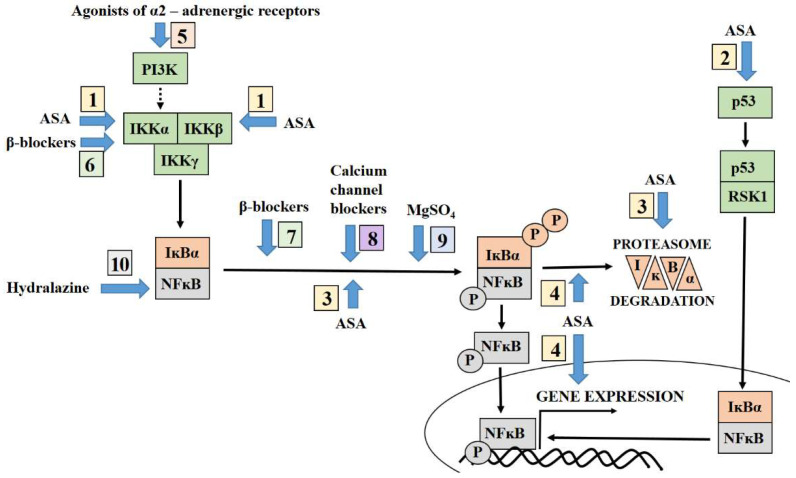
Targeting of NFĸB pathways by drugs adopted for the prevention and treatment of preeclampsia. Prevention of preeclampsia: (1–4) ASA (aspirin) is perceived as a negative regulator of NFkB, however, the mechanism by which it suppresses NFĸB activity varies between cell types. (1) In trophoblastic cell lines, ASA was found to inhibit the activators of NFĸB i.e., IKKα or IKKβ or (2) its action was related with the downregulation of *p53* gene expression. (3) The inhibition of phosphorylation and/or degradation of NFĸB inhibitors i.e., IĸBα or IĸBβ by ASA was observed in endothelial as well as in trophoblastic and tumour cell lines. (4) In some cells, repression of NFĸB-driven gene expression was observed following IĸBα degradation and nuclear translocation of NFĸB. Treatment of preeclampsia: (5) Some α2-adrenergic receptor agonists are perceived as activators of NFĸB via phosphoinositide-3-kinase (PI3K). (6, 7) β-blockers demonstrate different mechanisms of action in the regulation of NFĸB activity between cells; in lymphocytes, β-blockers act as inhibitors of NFĸB and repress the activation of IKKα (6), while in an endothelial cell line, they participate in the activation of NFĸB, favouring the phosphorylation and degradation of its inhibitor (7). NFĸB nuclear translocation is inhibited by attenuation of IĸBα phosphorylation by some calcium channel blockers (8) as well as MgSO_4_ i.e., magnesium sulphate (9). (10) Hydralazine downregulates the NFĸB protein level in cells.

Many of the drugs used for the prevention and treatment of preeclampsia are known to influence NFĸB activity (Figure 1). The most commonly-used drug to prevent preeclampsia is aspirin, an inhibitor of NFĸB activity. However, its mechanism of action varies between cells. While it inhibits NFĸB activators such as IKKα or IKKβ or influences the *p53* gene expression level in trophoblastic cells, it is known to regulate the phosphorylation and/or degradation of NFĸB inhibitors, i.e., IĸBα or IĸBβ, in other cell types, thus controlling the translocation of NFĸB into the nucleus.

Similarly, the drugs used to treat preeclampsia or prevent its neurological complications, such as sulphate magnesium, also are recognized as a modulators of NFĸB activity. Generally they inhibit the NFĸB/DNA binding reaction by attenuating phosphorylation followed by degrading IĸBα and nuclear translocation of NFĸB; some of them e.g., hydralazine, might also negatively regulate NFĸB level in cells.

Considering that NFĸB is perceived as the most important factor in preeclampsia development, and the drugs adopted for prevention and treatment of preeclampsia generally downregulate the activity of NFĸB, it is unclear why these drugs are not effective. It is possible that NFĸB activation pathways that are inhibited by drugs used in pregnancy are not preferred, and there are other mechanisms of NFĸB activation appearance in preeclampsia. Therefore, there is a need to better understand how (i.e., by what pathways) NFĸB activity is regulated at various stages of preeclamptic pregnancy. This issue seems to be especially important for selection or design of drugs that are planned for use in prevention of preeclampsia.

Pharmacological prevention of preeclampsia is begins before the onset of symptoms of disease, during the intensive development of the placenta, and is maintained for at least a few weeks of gestation. As a consequence, these drugs should have a time to restore the correct levels of NFĸB activity in the cells. Therefore, the further studies are necessary to determine: (1) whether drugs used in the prevention of preeclampsia should be mainly focused on regulating NFĸB activity in placental cells, (2) the NFĸB activation pathway that should be targeted by the new agents, and (3) the optimal point in pregnancy to include these drugs in therapy. Such knowledge will doubtlessly be beneficial in the development of new drugs and innovative methods of treatment of preeclampsia.

## Figures and Tables

**Table 1:** Recommendations given by selected organisations regarding ASA supplementation as preeclampsia prophylaxis among moderate and high-risk groups *.

Selected Word Organisation (Year)	ASA Dose	Initiation ASA Supplement	Bibliograph
World Health Organisation (WHO) (2011)	75 mg/day	<week 20	[35]
German Society of Gynaecology and Obstetrics (DGGG) (2015)	100 mg/day	no data	[36]
French Society of Cardiology (FESC)/French Society of Hypertension (2016)	75–160 mg/day	<week 20	[37]
The American College of Obstetricians and Gynaecologists (ACOG) (2018)	81 mg/day	week 12–28 Optimum <16	[38]
European Society of cardiology (ESC)/European Society of Hypertension (ESH) (2018)	100–150 mg/day	week 12	[39]
New Zealand Committee of the Royal Australian & New Zealand College of Obstetricians & Gynaecologists (RANZCOG) New Zealand College of Midwives (NZCOM) (2018)	≥75 mg/day optimum 100 mg/day	week 12	[40]
The International Society for the Study of Hypertension in Pregnancy (ISSHP) (2018)	75–162 mg/day	<week 20 Optimum <16	[41]
International Federation of Gynaecology and Obstetrics (FIGO) (2019)	150 mg/day (at night)	week 11–14	[42]
National Institute for Health and Care Excellence (NICE) (2019)	75–150 mg/day **	week 12	[43]
Polish Society of Hypertension (PTNT), Polish Cardiac Society (PTK) and Polish Society of Gynaecologists and Obstetricians (PTGiP) (2019)	100–150 mg/day	<week 16	[44]
International Society of Hypertension (ISH) (2020)	75–162 mg/day	week 12	[45]
Society for Maternal-Foetal Medicine (SMFM) (2020)	81 mg/day	week 12–28 Optimum <16	[46]

* The mentioned risk factors may vary slightly between organisations. Generally, high risk is associated with a history of preeclampsia, an adverse outcome in a previous pregnancy; expecting more than one child; chronic hypertension or with type 1 or 2 diabetes mellitus; renal disease; or autoimmunological disorders. Moderate risk is associated with more than one of the following factors: nulliparity (first pregnancy), age over 35–40 years, obesity, first degree family history of preeclampsia, low socioeconomic status, personal history of low birthweight, previous adverse pregnancy outcome or an interval of more than 10 years between pregnancies. ** Although this use is common in UK clinical practice, aspirin did not have marketing authorisation in the UK for this indication.

## Data Availability

Not applicable.
